# Spelling Performance of Portuguese Children: Comparison Between Grade Level, Misspelling Type, and Assessment Task

**DOI:** 10.3389/fpsyg.2020.00547

**Published:** 2020-03-27

**Authors:** Sofia Magalhães, Ana Mesquita, Marisa Filipe, Andreia Veloso, São Luís Castro, Teresa Limpo

**Affiliations:** Faculty of Psychology and Educational Sciences, University of Porto, Porto, Portugal

**Keywords:** Portuguese, spelling, misspellings type, grade comparison, assessment task

## Abstract

There is consensus among researchers that misspellings are something to avoid. However, misspellings also convey relevant information for researchers and educators. The present study is a first effort toward the analysis of misspellings produced by Portuguese children. Specifically, we aimed to examine the association between misspellings in dictation and composing tasks; compare misspellings across grade, type, and task; and test the contribution of different misspellings produced in dictation and in composition to text quality. For that, 933 Portuguese pupils in Grade 2 (*n* = 297), Grade 4 (*n* = 302), and Grade 6 (*n* = 334) performed a spelling-to-dictation task and wrote an opinion essay. Misspellings were categorized into phonetically inaccurate, phonetically accurate, and stress mark errors. Results showed correlations between the same type of misspellings across tasks for phonetically inaccurate errors in Grades 2 and 4, and phonetically accurate errors in Grade 2. Moreover, pupils produced more misspellings in dictation than composing tasks, and there was a progressive decrease in phonetically inaccurate and phonetically accurate misspellings across schooling, though stress mark errors were more frequent in Grade 4 than in other grades. Finally, spelling errors predicted text quality, particularly in younger children. Overall, these findings are aligned with extant results on spelling development and support current voices claiming for fine-grained analyses of misspellings. As they may vary across grade and task, and impact text quality differently, a detailed approach to spelling errors can provide valuable information on the development of this skill.

## Introduction

The importance of producing accurate spellings is undeniable. Problems with word spelling hamper readers’ comprehension, denote poor writing ability, and divert writers’ attention from other processes (Graham and Santangelo, 2014). In alphabetic writing systems, accurate spellings rely on a solid knowledge of phoneme-to-grapheme and orthographic conventions^1^ (Treiman and Bourassa, 2000). Good spellers need to be able to match speech sounds in a language (phonemes) with their accurate representation in written form (graphemes). Moreover, they need to master the orthographic constraints imposed by the orthographic depth of the language, which comprises the complexity and unpredictability of phoneme-grapheme correspondences (Schmalz et al., 2015). Orthographic depth varies along a continuum from shallow orthographies – with simple and consistent phoneme-grapheme relations – to deep orthographies – with complex and inconsistent sound-letter mappings (Katz and Frost, 1992). Concerning the orthographic depth of sound-to-print correspondences, European Portuguese has several phonemes with multiple representations (Lurdes de Castro, 2000), such as the phoneme /z/ that can be spelled ⟨z⟩, ⟨s⟩ or ⟨x⟩. These multiple correspondences make the learning of spelling challenging as reflected in the amount of misspellings produced by beginning writers (Mesquita et al., 2020). However, few studies examined the type of spelling errors produced by speakers of European Portuguese. The present study conducted an analysis of Portuguese children’s misspellings in order to increase understanding about failures in implementing basic word spelling procedures at different developmental stages; and to test whether such failures may be influenced by the spelling task and have impact on composing text.

According to a dual-route model (Barry, 1994), there are two procedures to spell a word: a phonological route that relies on sound-to-spelling conversions (assembled spelling) and a lexical route that retrieves known words from memory (addressed spelling). Grounded on this model, developmental theories proposed that the two routes are acquired successively, with children progressing from a partial-to-full *alphabetic phase* largely characterized by assembled spelling, to an *orthographic phase* that starts using addressed spelling (e.g., Ehri, 1986). Raising some objections to these theories, researchers recommended an approach to spelling development “as consisting of the predominant use of a particular process or strategy at different points in time, but not to the complete exclusion of others” (Treiman and Bourassa, 2000, p. 4). Beginning spellers may prioritize sound-based information, but they already rely on some orthographic knowledge; however, it is only with experience, that they become able to use multiple strategies to spell complex words (Cassar and Treiman, 1997). These claims have been supported by research examining spelling correctness of words with varying orthographic constraints (Defior et al., 2009). A study with Portuguese children found high accuracy rates in spelling words with unambiguous and context-dependent phoneme-grapheme mappings in Grade 2 (90 and 82%), though these latter were only mastered in Grade 4 (Mesquita et al., 2020). Similar findings were observed in Brazilian children (Pinheiro, 1995).

Common to many categorization systems of misspellings is the assumption that spelling is phonologically mediated (Treiman et al., 2019). Based on the dual-route model, a valuable classification of spelling errors, which was used in this study, is phonetically inaccurate vs. phonetically accurate (Treiman and Bourassa, 2000). In phonetically inaccurate errors, there is a mismatch between the spelling of the word and how it sounds [spelling the word ⟨ca**s**a⟩ (*house*) /′ka.zɐ / as ⟨ca**ss**a⟩/′ka.sɐ / results in a pronunciation that does not match the intended word]. In phonetically accurate errors, the phonological structure of the word is preserved, but an inappropriate orthographic interpretation is used (spelling ⟨ca**s**a⟩ or ⟨ca**z**a⟩ results in the same pronunciation /′ka.zɐ /, but the second form is orthographically incorrect). These errors may signal difficulties in using the spelling routes. Phonetically inaccurate errors indicate that spellers are not successfully using sound-based strategies, whereas phonetically accurate errors suggest a correct use of the assembled route, but a failure in using a lexical-based orthographic procedure. From a developmental stand, it is thus not surprising that phonetically inaccurate errors decrease throughout schooling and that the bulk of misspellings are phonetically accurate (Bahr et al., 2012; Protopapas et al., 2013).

This dichotomic classification of misspellings is, however, not without limitations (Moats, 1993; Bosman and Van Orden, 1997). Among others, it is not sensitive to specific complexities of some orthographic codes, such as the use of marks to indicate stress. Though related to phonology, their use is not governed by phoneme-to-grapheme correspondences, but rather by orthographic rules and lexical-level prosodic knowledge (Defior et al., 2012; Gutiérrez-Palma et al., 2019). In European Portuguese, there are three stress marks (acute accent /´/; circumflex accent /^∧^/; and tilde /∼/); for example, children learn that a stressed antepenultimate syllable requires an acute or circumflex accent to open or close the vowel, respectively (⟨p**ê**ndulo⟩ [*pendulum*] /′pẽ.du.lu/). Stress marks pose difficulties to Portuguese (Mesquita et al., 2020) as well as Spanish (Defior et al., 2009) and Greek (Protopapas et al., 2013) learners. Still, little is known about its incorrect use, including additions (⟨c**ô**xo⟩ for ⟨c**o**xo⟩ [*lame*] /′ko.ʃu/), omissions (⟨j**u**ri⟩ for ⟨j**ú**ri⟩ [*jury*] /′ ʒu.ɾi/), or substitutions (⟨c**á**o⟩ for ⟨c**ã**o⟩ [*dog*] /′k~ɐ w/). This may be linked to their underrepresentation in current theoretical models, largely based on English spelling, which do not include diacritics. Therefore, following Gutiérrez-Palma et al. (2019), this study addressed stress mark errors as an independent type of misspellings with the goal of providing preliminary empirical evidence on their prevalence in Portuguese children’s writing and contribute to refine explanatory approaches to word spelling.

Another understudied aspect is the extent to which spelling is task dependent. Typically, spelling abilities are assessed in dictation or composing tasks that challenge spellers differently. In dictation tasks, participants are asked to spell pre-defined items chosen to assess specific features of the spelling system; in composing tasks, participants are asked to write a text in response to a specific prompt and they are free to choose the words to write, including to avoid those features. Moreover, in dictation tasks, participants’ only job is to retrieve, assemble, and select the word’s orthographic representation and write it down; in composing tasks, they also need to enact many other processes, such as ideation, translation, and reconceptualization (Graham, 2018). A handful of studies reported correlations between dictation and composing misspellings, from 0.25 in American to 0.71 in Italian pupils (Graham et al., 1997; Limpo and Alves, 2013; Bigozzi et al., 2016), indicating an overlap between tasks. Nonetheless, no information about the impact of the assessment task on the type of misspellings was provided.

Because of the many processes competing for writers’ attention during composition, being able to spell accurately is a valuable asset for young learners (Alves et al., 2018). Research showed that spelling skills constrain text quality (Graham et al., 1997; Abbott et al., 2010; Limpo and Alves, 2013), used as an indicator of writers’ ability to create texts with good and coherently organized ideas, conveyed through well-crafted sentences and interesting vocabulary (Cooper, 1997). As claimed by recent cognitive writing models (Graham, 2018), due to spelling difficulties during composition, poor spellers have limited resources for other processes (e.g., idea generation, language formulation). In spite of this claim, no study tested whether text quality is more affected by pupils’ difficulties with sound-based conversions (phonetically inaccurate errors), orthographic and/or lexical knowledge (phonetically accurate errors), or word stressing (stress mark errors).

## Present Study

Grounded on the dual-route model (Barry, 1994), we aimed to examine the success of Portuguese pupils in implementing sound- and orthographic-based spelling strategies. For that, we categorized misspellings into phonetically inaccurate or phonetically accurate errors (please see section “Procedure and Tasks”). A third category of errors was considered – stress marks – to examine whether word stressing was problematic for learners, as suggested before (Defior et al., 2009). Misspellings were compared across Grades, 2, 4, and 6 to study the evolution of each error type. Because stage theories (Ehri, 1986) suggest a progression from sound- to orthographic-based strategies, we expected more phonetically inaccurate errors in younger pupils, and an overall higher percentage of phonetically accurate and stress mark errors (Bahr et al., 2012). To test the premise that spelling skill is task dependent, we also compared the type of misspellings across two tasks with varying constraints (dictation vs. composing). Besides moderate between-tasks correlations (Limpo and Alves, 2013; Bigozzi et al., 2016), we anticipated that, despite the greater demands of composing, this task would elicit less errors, by allowing participants to choose the words to write (Graham et al., 1997; Limpo and Alves, 2013; Bigozzi et al., 2016). Finally, we aimed to deepen past findings showing contributions of spelling to writing (Graham et al., 1997; Abbott et al., 2010; Limpo and Alves, 2013) by identifying the type of misspellings with the strongest impact on text quality. We hypothesized that phonetically inaccurate errors, as markers of a failure in the basic mechanism to spell words (assembled spelling; Barry, 1994), would have the most damaging impact on writing. Together, these findings may improve our understanding of spelling development and provide useful hints to inform assessment and instructional spelling practices.

## Method

### Participants

Participants were 933 Portuguese native speakers in Grades 2, 4, and 6, who came from 50 classes from five public clusters of schools, holding collaboration protocols with authors’ University. All pupils attending school in the data collection day and without special education needs were included. The sample comprised 297 second graders (*M*_*age*_ = 7.68 years, *SD* = 0.37; 44% girls), 302 fourth graders (*M*_*age*_ = 9.72 years, *SD* = 0.39; 52% girls), and 334 sixth graders (*M*_*age*_ = 11.66 years, *SD* = 0.43; 55% girls). For characterization purposes, we surveyed pupils’ grades in core subjects (Portuguese and Mathematics) and mothers’ educational level. Overall, our sample presented values slightly above the general population, as detailed in Supplementary Table S1.

### Instructional Setting

Spelling has a central role in the Portuguese primary writing curriculum (Buesco et al., 2015). In Grades 1–2, spelling instruction is greatly focused on conveying basic phonological and orthographic knowledge. Children learn sound-to-print correspondences and consistent orthographic features (e.g., digraphs and context-dependent mappings). From Grade 3 onward, explicit spelling instruction is reduced, with the focus of writing instruction being on composing situations, where children should consolidate difficult orthographic complexities (e.g., inconsistencies or diacritics).

### Procedure and Tasks

In one 30-min session, classroom groups with 20–25 pupils composed an opinion essay and spelt 16 words dictated at 10-s intervals.^2^

The procedure for the composing task was similar across grades. Pupils had 10 min to write the text, and they were notified 5 min and 1 min before the end of the time limit. Essay topics were: “Do you think children should eat candies whenever they want?” for Grade 2, “Do you think pupils’ should have more field trips?” for Grade 4, and “Do you think teachers should give pupils homework every days?” for Grade 6. These prompts were previously identified by primary- and middle-grade schoolteachers as appropriate for pupils of the respective grades in terms of difficulty and interest value, thereby maximizing task engagement and productivity.

The 16-word list comprised four words from four orthographic complexity categories of the Portuguese spelling system, namely, consonant clusters (e.g., ⟨te**cl**ado⟩ *keyboard* /tε.′kla.du/), stress marks (e.g., ⟨j**uì**ri*⟩ jury* /′ʒu.ɾi/), inconsistencies (e.g., ⟨**g**ema⟩ *yolk* /′ʒe.mɐ /), and silent ⟨h⟩ (e.g., ⟨hino⟩ *anthem* /′i.nu/). These words were selected from a 56-word test used in previous research (Limpo and Alves, 2013; Alves and Limpo, 2015; Mesquita et al., 2020), which time-related reasons prevented us to use here. The 16 words were selected by excluding complexity categories with high accuracy rates in Grade 2 and words less sensitive to grade level. Based on non-published data from Alves and Limpo (2015), performance in the 56- and 16-word list was strongly correlated (*r* = 0.84). The list here used includes bi- and trisyllable words of 4-to-7 letters, roughly half of them of high frequency and the other half of low-to-medium frequency (more information on the 16 words appears in Supplementary Table S2, and the 56-word test is described in Mesquita et al., 2020).

### Measures

#### Spelling Errors

The number and type of spelling errors was examined in the dictation and composing tasks. Misspellings were counted and categorized into three types: phonetically inaccurate (e.g., spelling ⟨gema⟩ /′ʒe.mɐ /as ⟨**x**ema⟩ /′∫e.mɐ /), phonetically accurate (e.g., spelling ⟨gema⟩ as ⟨**j**ema⟩, both forms accurately sound as /′∫e.mɐ /), and stress mark (surplus, omission, or substitution of diacritics; e.g., spelling ⟨gema⟩ as ⟨g**ê**ma⟩). Due to lack of legibility, the spelling correctness of 1% if the words could not be discerned. Given the reduced percentage, these words were not considered in further analyses. The final score for both tasks was the percentage of each error type, computed by dividing number of errors by number of words dictated or written in the essay. By using percentages, we accounted for differences in the amount of words produced in the compositions: 41 (*SD* = 28), 52 (*SD* = 29), and 54 (*SD* = 29) in Grades 2, 4, and 6, respectively. To allow the computation of percentage in reference to total words, for words with several errors only one was counted, following a hierarchy of error severity from readers viewpoint, being in decreasing order phonetically inaccurate, phonetically accurate, and stress mark.

#### Text Quality

The quality of opinion essays was rated by two trained graduate research assistants with an holistic scale (based on Cooper, 1997). Raters gave a single value to each text from 1 (*low quality*) to 7 (*high quality*), taking ideas quality, organization, sentence structure, and vocabulary into account (Limpo and Alves, 2013). To control for expected grade differences, texts were grouped and rated separately by grade. Judges were then provided with representative examples of low-, medium-, and high-quality texts within each grade level (for a similar procedure see Graham et al., 1997; Alves and Limpo, 2015). To avoid biased judgments, all texts were typed and corrected for misspellings (Berninger and Swanson, 1994). The final score was the average across judges.

#### Reliability

Spelling measures from 80 pupils per grade (25–30%) were rescored by a second judge. Interrater agreement measured with the intraclass correlation coefficient (ICC) for single measures and separately by grade was above 0.82 and 0.92 for misspellings in composing and dictation, respectively. Because text quality of all participants was double scored, we computed average measures ICC, which was above 0.92.

## Results

### Characterization of Pupils’ Misspellings

To characterize misspellings, we conducted a preliminary examination of descriptive statistics (Table 1) and correlations (Table 2) for misspellings by grade. Noteworthy findings were: correlations between the same type of misspellings in dictation and composition were observed for phonetically inaccurate errors in Grade 2 (*r* = 0.33) and Grade 4 (*r* = 0.25), and for phonetically accurate errors in Grade 2 (*r* = 0.25); correlations between different types of misspellings in dictation (namely, between phonetically inaccurate and phonetically accurate, between phonetically inaccurate and stress marks, and between phonetically accurate and stress marks errors) were evident mainly in Grade 2 (−0.18 < *r*s < −0.52), whereas correlations between different types of misspellings in composition were stronger in Grade 4 (0.27 < *r*s < 0.30); poorer texts were generally associated with more misspellings, particularly in second graders (−0.11 < *r*s < −0.26).

**TABLE 1:** Means and standard deviations of the misspellings produced in the dictation and composing tasks by grade.

	Grade 2		Grade 4		Grade 6	
	(*n* = 297)		(*n* = 302)		(*n* = 334)	
Measures	*M*	*SD*	*M*	*SD*	*M*	*SD*
Percentage of spelling errors in dictation (total)	72.85	16.77	47.25	15.96	33.31	14.15
Phonetically inaccurate	17.68	18.69	4.95	8.81	3.71	6.17
Phonetically accurate	40.80	14.01	26.26	11.03	16.60	6.12
Stress mark	12.23	8.65	15.94	8.25	12.43	9.14
Percentage of spelling errors in text (total)	13.05	10.96	11.31	16.40	1.73	5.92
Phonetically inaccurate	4.12	6.35	1.69	4.08	0.11	0.65
Phonetically accurate	6.18	6.68	4.25	8.57	0.64	1.62
Stress mark	2.63	3.86	5.29	9.41	0.67	1.67
Text quality (1–7)	3.10	1.38	3.24	1.32	3.54	1.00

**TABLE 2:** Correlations among misspellings in the dictation and composing tasks by grade.

	Total		Phonetically inaccurate		Phonetically accurate		Stress mark		Text
	Dictation	Composing	Dictation	Composing	Dictation	Composing	Dictation	Composing	Quality
**Grade 2**									
*Total*									
Dictation		0.23	0.73	0.28	0.30	0.14	–0.41	–0.04	–0.21
Composing			0.15	0.64	0.17	0.74	–0.16	0.45	–0.30
*Phonetically inaccurate*									
Dictation				0.33	–0.18	0.02	–0.52	–0.14	–0.18
Composing					–0.08	0.09	–0.21	0.01	–0.26
*Phonetically accurate*									
Dictation						0.25	–0.26	0.18	–0.11
Composing							–0.10	0.16	–0.16
*Stress mark*									
Dictation								–0.06	0.14
Composing									–0.14
**Grade 4**									
*Total*									
Dictation		0.22	0.58	0.27	0.71	0.15	0.34	0.12	–0.07
Composing			0.20	0.57	0.07	0.75	0.09	0.80	–0.29
*Phonetically inaccurate*									
Dictation				0.25	0.14	0.17	–0.17	0.08	–0.15
Composing					0.17	0.27	0.02	0.30	–0.22
*Phonetically accurate*									
Dictation						0.04	–0.11	0.02	–0.06
Composing							0.04	0.28	–0.22
*Stress mark*									
Dictation								0.12	0.10
Composing									–0.20
**Grade 6**									
*Total*									
Dictation		0.09	0.57	0.09	0.55	0.17	0.73	0.22	–0.18
Composing		0.02	0.11	0.12	0.27		0.04	–0.26	–0.14
*Phonetically inaccurate*									
Dictation				0.07	0.12	0.06	0.09	0.11	–0.16
Composing					0.05	0.08	0.07	–0.06	–0.03
*Phonetically accurate*									
Dictation						0.06	0.11	–0.12	–0.03
Composing							0.18	0.03	–0.07
*Stress mark*									
Dictation								0.19	–0.11
Composing									–0.03

*Correlations of 0.12 or above for Grades 2 and 4, and of 0.11 or above for Grade 6 are significant at an alpha level of 0.05.*

### Comparison of Misspellings Across Grade, Type, and Task

**FIGURE 1:**
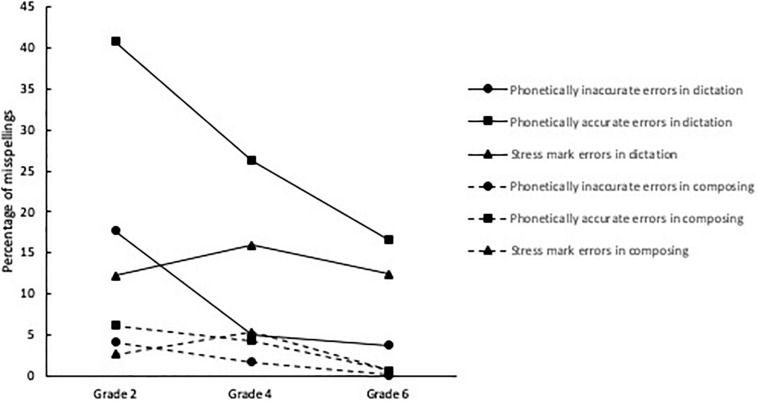
Illustration of the Grade × Error Type X Spelling Task interaction.

We conducted a 3 (Grade [Grade 2, Grade 4, Grade 6]) x 3 (Misspelling type [phonetically inaccurate, phonetically accurate, stress mark]) x 2 (Task [composing, dictation]) ANOVA with repeated measures on the last factors. Results revealed three main effects: Grade, *F*(2,930) = 440.70, *p* < 0.001, η_*p*_^2^ = 0.49; Misspelling type, *F*(2,929) = 649.27, *p* < 0.001, η_*p*_^2^ = 0.58; and Task, *F*(1,930) = 5385.38, *p* < 0.001, η_*p*_^2^ = 0.85; and three 2-way interactions: Grade x Misspelling type, *F*(4,1860) = 111.29, *p* < 0.001, η_*p*_^2^ = 0.19; Grade x Task, *F*(2,930) = 204.60, *p* < 0.001, η_*p*_^2^ = 0.31; and Misspelling type x Task, *F*(2,929) = 555.74, *p* < 0.001, η_*p*_^2^ = 0.55. We also found a significant 3-way interaction, *F*(4,1860) = 64.17, *p* < 0.001, η_*p*_^2^ = 0.12, illustrated on Figure 1 and decomposed with simple effects analyses described below.

#### Differences Between Grade Levels

We found grade differences for all error types in both tasks, *F*s(2,930) > 17.55, *p*s < 0.001, η_*p*_^2^ > 0.04. Pairwise comparisons showed that the percentage of phonetically inaccurate and phonetically accurate errors in both tasks was significantly different among the three grades (*p*s < 0.001, except for differences in the percentage of phonetically inaccurate errors in the dictation task between Grade 4 and 6, *p* = 0.20), with second graders displaying more errors and sixth graders displaying less errors. The pattern of findings was different for stress mark errors: in composition, fourth graders produced more errors than second graders, who in turn produced more errors than sixth graders (*p*s < 0.001); in dictation, fourth graders produced more errors than both second and sixth graders (*p*s < 0.001), who did not differ one another (*p* = 0.78).

#### Differences Between Misspelling Types

Except for Grade 6 in the composing task, where the percentage of misspellings did not vary across error type (*p* = 0.22), we found differences in error types for both tasks and in the three grades, *F*s(2, 929) > 33.43, *p*s < 0.001, η_*p*_^2^ > 0.07. For the composing task, the percentage of errors in decreasing order was phonetically accurate, phonetically inaccurate, and stress mark in Grade 2, and stress mark, phonetically accurate, and phonetically inaccurate in Grade 4; for the dictation task, the percentage of errors in decreasing order was phonetically accurate, phonetically inaccurate, and stress mark in Grade 2, and phonetically accurate, stress mark, and phonetically inaccurate in Grades 4 and 6 (*p*s < 0.02).

#### Differences Between Spelling Tasks

We found task differences for all error types in the three grades, *F*s(1, 930) > 23.33, *p*s < 0.001, η_*p*_^2^ > 0.02, with consistently more errors in dictation than composition.

### Contribution of Misspellings to Text Quality

To examine the contribution of misspellings to text quality, we conducted regression analyses for each grade (Table 3). In Grade 2, misspellings explained 12% of the variability in text quality, *R* = 0.35, *F*(6, 290) = 6.76, *p* < 001. Significant predictors were phonetically inaccurate errors in the dictation task (*b* = −0.16) and phonetically inaccurate (*b* = −0.21) and stress mark (*b* = −0.13) errors in the composing task. In Grade 4, misspellings explained 11% of the variability in text quality, *R* = 0.33, *F*(6,295) = 9.50, *p* < 001. Significant predictors were stress mark errors in the dictation task (*b* = 0.12) and phonetically inaccurate (*b* = −0.12), phonetically accurate (*b* = −0.14), and stress mark (*b* = −0.13) errors in the composing task. In Grade 6, misspellings explained 4% of the variability in text quality, *R* = 0.20, *F*(327,6) = 2.22, *p* = 0.04, with phonetically inaccurate errors in dictation (*b* = −0.15) being the unique predictor.

**TABLE 3:** Parameter estimates for models regressing text quality onto misspellings by grade.

Predictors	*B*	*SE*	*b*	*t*	*p*
**Grade 2**					
*Misspellings in dictation*					
Phonetically inaccurate	–0.01	0.01	–0.16	–2.30	0.02
Phonetically accurate	–0.01	0.01	–0.12	–1.81	0.07
Stress mark	<0.001	0.01	–0.03	–0.35	0.07
*Misspellings in composing*					
Phonetically inaccurate	–0.05	0.01	–0.21	–3.62	<0.001
Phonetically accurate	–0.02	0.01	–0.08	–1.33	0.19
Stress mark	–0.05	0.02	–0.13	–2.29	0.02
**Grade 4**					
*Misspellings in dictation*					
Phonetically inaccurate	–0.01	0.01	0.07	–1.17	0.24
Phonetically accurate	<0.001	0.01	<0.001	–0.07	0.95
Stress mark	0.02	0.01	0.12	2.05	0.04
*Misspellings in composing*					
Phonetically inaccurate	–0.04	0.02	–0.12	–1.99	0.05
Phonetically accurate	–0.03	0.01	–0.14	–2.38	0.02
Stress mark	–0.02	0.01	–0.13	–2.23	0.03
**Grade 6**					
*Misspellings in dictation*					
Phonetically inaccurate	–0.02	0.01	–0.15	–2.71	0.02
Phonetically accurate	0.00	0.01	<0.001	–0.04	0.97
Stress mark	–0.01	0.01	–0.10	–1.83	0.07
*Misspellings in composing*					
Phonetically inaccurate	<0.001	0.09	<0.001	0.00	1.00
Phonetically accurate	–0.03	0.04	–0.04	–0.67	0.50
Stress mark	0.04	0.03	–0.06	1.10	0.27

## Discussion

This study aimed to examine the types of misspellings in spelling-to-dictation and composing tasks produced by second-, fourth-, and sixth-grade Portuguese pupils.

An examination of the correlations between the same type of misspellings in dictation and composition showed that the percentage of phonetically inaccurate errors in Grades 2 and 4, and of phonetically accurate errors in Grade 2 which were produced in dictation was associated with that produced in composition. This finding partially agrees with other studies showing correlations between misspellings in dictation and composing tasks (e.g., Graham et al., 1997; Limpo and Alves, 2013; Bigozzi et al., 2016). However, the low correlations in Grades 2 and 4 (<0.33) and general lack of correlation in Grade 6 (<0.19) seem to reflect the different conditions under which spelling is measured. In dictation, pupils’ major task is to spell isolated words, whereas in composition, processes other than these compete for writers’ attention (e.g., idea generation; Graham, 2018). Additionally, in the dictation task, participants were forced to spell a set of pre-defined words, some of them with very low frequency of occurrence and representing difficult orthographic features of the Portuguese spelling system (e.g., consonant cluster, stress marks, and phoneme-grapheme inconsistencies). In the composing task, children were free to choose the words they wanted to write.

These differences between the dictation and composing tasks can also explain the finding that, consistently across grades and types of misspellings, the dictation task resulted in more spelling errors than the composing task. This finding was not surprising. Given the low percentage of misspellings (average of 8% for the whole sample), children seemed very effective in selecting words they knew how to spell (Graham and Santangelo, 2014). Actually, in Grade 6, the percentage of misspellings in composition was below 2%, suggesting that pupils become increasingly strategic throughout schooling. However, this finding also indicates that composing tasks might not be a sensitive indicator of older pupils’ spelling skill. Composition-based measures may mask spelling’ difficulties and provide a biased picture of writers’ abilities.

It should additionally be noted that because of the forced vs. free selection of words that characterizes dictation and composing tasks, the type of misspelled words compared was probably different. This may be another factor contributing to the above-discussed inter-task differences concerning correlational patterns and percentage of misspellings. For example, can the same word spelled in dictation and composing tasks be similarly misspelled? For a stringent test to the effects of assessment task on misspellings, future research should manipulate dictation and composing tasks to elicit comparable words. This could be achieved by using lists composed of words either closer to those that children produce and are exposed to in school, or in line with the topic of composition.

In general, pupils in higher grades produced less phonetically inaccurate and phonetically accurate misspellings than those in lower grades. With experience and instruction, children acquire new strategies and knowledge that allows them to produce less and less misspellings (Treiman and Bourassa, 2000). This developmental pattern is not new (Bahr et al., 2012; Alves and Limpo, 2015), but it provides relevant practical indications. Despite the decrease, sixth graders failed to correctly spell 33% of the words dictated. Indeed, though explicit spelling instruction in Portuguese schools seems to occur only in primary years, pupils show evidence of not having mastered the complexities of the Portuguese spelling system during that period. This skill should perhaps be explicitly taught and systematically practice until difficult orthographic features are fully learned. For a deeper understanding of the specific features that are a struggle for pupils at different grades, future studies should include fine-grained analyses of misspellings at the stimulus level.

The decreasing trend observed for phonetically inaccurate and phonetically accurate misspellings was, however, not observed for stress mark errors, which were more frequent in Grade 4. Unexpectedly, the more frequent these errors were in the dictation task, the better the quality of fourth graders compositions. The present study does not provide compelling explanations for these findings. Though they may represent a sample artifact, they may also be linked to the way stress marks were taught to the children observed here. The relationship between instructional practices and pupils’ performance, particularly in terms of stress assignment, should receive further research attention. Past studies already indicated that children struggle with the learning of this spelling feature in particular (e.g., Defior et al., 2009). Stress mark errors may signal poor knowledge of lexical stress and difficulties in mapping orthography and prosody (Defior et al., 2012; Gutiérrez-Palma et al., 2019). A question still to be answered is how this information is being taught in primary grades. At least for Portuguese spellers there are no evidence-based practices that teachers can use to foster pupils’ knowledge about the appropriate placement of stress marks in words.

Considering all pupils together, stress mark and phonetically accurate errors represented the majority of misspellings produced in both tasks (cf. Bahr et al., 2012). In comparison, phonetically inaccurate errors were less frequent, confirming that the learning of sound-based spelling strategies occurs in the earliest phases of spelling development (Ehri, 1986; Treiman and Bourassa, 2000). In dictation, phonetically accurate errors were consistently higher than phonetically inaccurate errors, suggesting an overall success in using the assembled route to spell words, but a less-than-desirable ability in using the orthographic-based procedure. From an applied viewpoint, this means that spelling instruction is not being entirely successful in fostering addressed spelling. Past research already showed the differential benefits of varying training methods to improve spelling accuracy (Berninger et al., 1998; Van Leerdam et al., 1998). Future research should complement these findings by looking at the effectiveness of those methods to suppress specific types of misspellings, in particular those resulting from failures in the orthographic processing system.

We found an effect of spelling on text quality that supports theoretical claims (Berninger and Winn, 2006; Graham, 2018) and replicates past findings (Abbott et al., 2010; Limpo and Alves, 2013; Limpo et al., 2017). Misspellings explained 12, 11, and 4% of the variance in text quality in Grades 2, 4, and 6, respectively. This low percentage was not unexpected. It aligns with writing models proposing that spelling is a key writing process (Graham, 2018), alongside many others not here examined (e.g., idea generation, language formulation, reviewing, executive functions). Furthermore, older pupils’ spelling seemed to play a smaller role in writing, supporting the claim that throughout schooling as spelling gets more automatic and interferes less with composing quality (Graham et al., 1997; Berninger, 1999; Abbott et al., 2010).

This study also showed the specificity of the misspellings’ effects on text quality. We found that poorer texts were associated with (a) phonetically inaccurate errors in dictation and composition, and stress mark errors composition in Grade 2; (b) stress mark errors dictation and all types of errors in composition in Grade 4; (c) phonetically inaccurate errors in dictation in Grade 6. As already anticipated from the correlation analyses, it seems that neither all types of misspellings interfered with text quality nor to the same degree, suggesting the involvement of varying levels of attentional resources in different word spelling processes. At least here, the most consistent predictors were phonology-based misspellings. These errors may indicate lack of automaticity in sound-to-print conversions, which may need extra attentional resources that are diverted from other processes underlying good writing. Pupils did not produce this type of errors very often – though it represented 18% of the second graders’ misspellings in dictation – but those who did it, seem at a clear disadvantage. Teachers should be sensitive to their occurrence in any grade and implement either preventive or remediating practices to eliminate them.

Moreover, the finding that phonetically accurate errors were generally unrelated to text quality may indicate that pupils do not seem to struggle with the addressed spelling route, even when disrupted. For example, little interference in writing is expected if pupils are not aware of an orthographic rule and believe to be spelling correctly (which is reinforced by the fact that the misspelled word sounds as the intended word). It is worth noticing that the holistic measure of text quality prevented us to ascertain the specific text features (e.g., discourse, sentence, word) affected (or not) by different types of misspellings, which can be done by employing analytic measures. These findings also imply that depending on children’s grade, some tasks maybe more appropriate than others to uncover the link between spelling and text quality. Composing tasks seem useful to assess spelling skills and examine its predictive value in younger pupils; whereas they seem less valuable in older pupils, who may act strategically as previously noted.

## Conclusion

There is no question that misspellings are something to avoid. However, as suggested by current and other findings (e.g., Treiman et al., 2019), misspellings also convey key information for researchers and educators. Despite the proved value of looking into spelling accuracy (Abbott et al., 2010), examining the type of misspellings provides fine-grained data that may not only deepen researchers’ knowledge about learning to spell and its role on writers’ ability to produce text, but also inform educators about the most suitable instructional practices to fulfill pupils’ writing needs.

## Data Availability Statement

The datasets generated for this study are available on request to the corresponding author.

## Ethics Statement

The studies involving human participants were reviewed and approved by Faculty of Psychology and Educational Sciences of the University of Porto. Written informed consent to participate in this study was provided by the participants’ legal guardian/next of kin.

## Author Contributions

TL and SC designed the study. SM, AM, MF, and AV collected and coded the data. TL analyzed and interpreted the data. All authors wrote and reviewed the manuscript and approved its final version.

## Conflict of Interest

The authors declare that the research was conducted in the absence of any commercial or financial relationships that could be construed as a potential conflict of interest.

## Footnotes

1 Children learning alphabetic scripts need to master phoneme-to-grapheme (spelling) and grapheme-to-phoneme (reading) correspondences, which are undoubtably related (Shankweiler and Lundquist, 1993). However, since spelling is the main topic of this brief report, for the sake of concision, only phoneme-to-grapheme correspondences are addressed.

2 With the initial purpose of testing the contribution of misspellings to text quality above and beyond handwriting skill (i.e., ability to produce fast and accurate handwriting), pupils were also asked to perform two handwriting fluency tasks. However, introducing handwriting into the regression models led to the exact same results (see section “Contribution of Misspellings to Text Quality”). Thus, for the sake of parsimony, this variable was not included in any analysis and therefore not addressed in the manuscript.
