# Supplementary material for: Spelling Performance of Portuguese Children: Comparison Between Grade Level, Misspelling Type, and Assessment Task
Source: Front Psychol. 2020 Mar 27;11:547. doi: 10.3389/fpsyg.2020.00547 (PMC7120319; doi:10.3389/fpsyg.2020.00547)
Supplement: Supplementary file 1 [file Data_Sheet_1.PDF]

Supplementary Material

1 Supplementary Table S1: Participants' Characterization by Grade.

Note. Pupils' grades in the school subjects of Portuguese and Mathematics were collected as an indicator of their school achievement. These grades are attributed by their respective teachers in a scale ranging from 1 (*lowest score*) to 5 (*highest score*). Though national information concerning primary school results is unavailable, data targeting middle school in 2014/15 indicated that school achievement of our sample might be slightly above national results. In Grade 6, Portuguese and Math average grades of pupils attending public schools only were 3.32 (*SD* = 0.74) and 3.08 (*SD* = 0.98) (DGEEC, 2017). The educational level of pupils' mothers was used as an indicator of their socioeconomic status. As can be seen in the Table, the three grades presented similar values. However, data concerning the whole Portuguese people revealed that the mothers of our sample seem more highly educated. In 2019, Portuguese national statistics regarding females' educational level is as follows: 21.5% completed Grade 4; 27% completed Grade 9; 22% completed high school; 22% completed college or college plus some postgraduate studies; and 9% was unknow (Fundação Francisco Manuel dos Santos, 2020).

Variables	Grade 2	Grade 4	Grade 6
School grades (1-5)			
$M_{\text{Portuguese}}$ (SD)	3.82 (0.90)	3.60 (0.78)	3.42 (0.63)
$M_{\text{Mathematics}}$ (SD)	3.83 (0.89)	3.63 (0.89)	3.51 (0.67)
Mother's educational level (%)			
Grade 4	1.5	11	4
Grade 9	27	35	39
High School	34	34	25
Graduation or above	35.5	19	31.5
Unknown	2	1	0.5

References

Direção-Geral das Estatísticas da Educação e Ciência. (2017). *Resultados escolares por disciplina: 2º Ciclo - Ensino Público. Ano letivo 2014-2015 [School results by subject: 2º cycle, public teaching, academic year of 2014-2015*]. Lisboa: Direção-Geral das Estatísticas da Educação e Ciência. Retrieved from: http://www.dgeec.mec.pt/np4/369/%7B$clientServletPath%7D/?newsId=784&fileName=DGEEC_2017_ResultadosDisciplinas2_CEB2.pdf

Fundação Francisco Manuel dos Santos. (2020). *PORDATA, Base de dados Portugal contemporâneo* [PORDATA, Contemporary Portugal Data- base]. Retrieved from http://www.pordata.pt/

2 Supplementary Table S2: 16-Word List Used in the Spelling-to-Dictation Test.

Note. These words were selected from a test used in past studies (Alves & Limpo, 2015; Limpo & Alves, 2013; Mesquita et al., 2020), composed of 56 words representing seven orthographic complexities: digraph, contextual consistency, position consistency, consonant cluster, stress mark, inconsistency, and silent letter ‹h› (a thorough description of the test and its development appears in Mesquita et al., 2020). As time-related constrains prevented us to administer the full list, this had to be reduced. For that, we analysed the misspellings produced by the pupils in Alves and Limpo (2015) (data not published) and followed a two-step procedure to select the words. First, we removed words from categories with the highest accuracy rates in Grade 2 (> 75%; these categories were also the ones with the best performance in Mesquita et al., 2020, accuracy > 80% in Grade 2). Second, for each of the other four categories (consonant cluster, stress mark, inconsistency, and silent letter ‹h›) we selected the four words providing the best between-grade discrimination. After achieving the final 16-word list, we confirmed that the number of misspellings in the 16-word list was able to discriminate between grades (ps < .05), and held strong correlations with the number of misspellings in the 56-word list (> .84), indicating grade-level sensitivity of the reduced list and concurrent validity with the original one. The final list includes bi- and trisyllable words of 4-to-7 letters, with frequency per million for Grades 1-6, extracted from ESCOLEX (Soares et al., 2014), ranging from .031 to 109.91 (by complexity category, all words have an average F_{million} of about 30, except the words in the consonant cluster, which are fully consistent, and therefore chosen to be less frequent, average F_{million} = 9).

Words	Number of		F_{million}	Complexity category
	Letters	Syllables		
teclado	7	3	12.14	**Consonant cluster:** refers to consecutive consonants at the onset of a syllable (CCV syllable). In this category, the second consonant of the cluster was /ɾ/ or /l/.
drama	5	2	0.93	
sagrado	7	3	6.54	
dupla	5	2	16.50	
júri	4	2	5.29	**Stress marks:** indicate whether the vowel is open or closed through a rule-based placement of diacritics to indicate lexical stress.
fértil	6	2	11.52	
último	6	3	109.91	
pêndulo	7	3	4.36	
gema	4	2	14.94	**Inconsistency:** occurs when a phoneme-grapheme mapping is not predictable on the basis of contextual rules. For example, the phoneme /ʒ/ can be represented by ‹g› or ‹j› (e.g., ‹gema› *yolk* /'ʒe.mɐ/).
tigela	6	3	18.68	
lojista[a]	7	3	53.24	
anexo	5	3	28.96	
hino	4	2	30.51	**Silent ‹h›:** the letter ‹h› as a single grapheme is used in word initial position and has no phonemic value. For example, ‹hera› *ivy* and ‹era› *was/era* are both pronounced /'ɛ.ɾɐ/.
hípico	6	3	0.31	
haste	5	2	12.45	
humana	6	3	74.10	

[a]This word did not appear in the frequency database, thus F_{million} refers to the base word ‹loja› *store*, wich may represent a slight overestimation of the frequency of the target word ‹lojista› *store owner*.

References

Alves, R. A., & Limpo, T. (2015). Progress in written language bursts, pauses, transcription, and written composition across schooling. *Scientific Studies of Reading, 19*, 374-391. doi:10.1080/10888438.2015.1059838

Limpo, T., & Alves, R. A. (2013). Modeling writing development: Contribution of transcription and self-regulation to Portuguese students' text generation quality. *Journal of Educational Psychology, 105*, 401-413. doi:10.1037/a0031391

Mesquita, A., Carvalhais, L., Limpo, T., & Castro, S. L. (2020). Portuguese spelling in primary grades: Complexity, length and lexicality effects. *Reading and Writing: An Interdisciplinary Journal*. doi:10.1007/s11145-019-10012-5

Soares, A. P., Medeiros, J. C., Simões, A., Machado, J., Costa, A., Iriarte, A., . . . Comesaña, M. (2014). ESCOLEX: A grade-level lexical database from European Portuguese elementary to middle school textbooks. *Behavior Research Methods, 46,* 240-253. doi:10.3758/s13428-013-0350-1
